# Non-Invasive Prenatal Diagnosis in the Management of Preimplantation Genetic Diagnosis Pregnancies

**DOI:** 10.3390/jcm3030913

**Published:** 2014-08-14

**Authors:** Ana Bustamante-Aragones †, Sara Perlado-Marina †, Maria José Trujillo-Tiebas, Jesús Gallego-Merlo, Isabel Lorda-Sanchez, Luz Rodríguez-Ramirez, Concepcion Linares, Corazón Hernandez, Marta Rodriguez de Alba

**Affiliations:** 1 Genetics Service, Fundacion Jimenez Diaz-IIS, Avda. Reyes Catolicos, 2, 28040 Madrid, Spain; E-Mails: sara.perlado@fjd.es (S.P.-M.); mjtrujillo@fjd.es (M.J.T.-T.); jgallegom@fjd.es (J.G.-M.); ilorda@fjd.es (I.L.-S.); mrodrigueza@fjd.es (M.R.A.); 2 Assisted Reproduction Unit, Fundacion Jimenez Diaz-IIS, Avda. Reyes Catolicos, 2, 28040 Madrid, Spain; E-Mails: lrodriguezr@fjd.es (L.R.-R.); clinares@fjd.es (C.L.); chernandez@fjd.es (C.H.)

**Keywords:** ccffDNA, maternal plasma, NIPD, preimplantation genetic diagnosis

## Abstract

Prenatal diagnosis (PD) is recommended in pregnancies after a Preimplantation Genetic Diagnosis (PGD). However, conventional PD entails a risk of fetal loss which makes PGD patients reluctant to undergo obstetric invasive procedures. The presence of circulating fetal DNA in maternal blood allows performing a non-invasive prenatal diagnosis (NIPD) without risk for the pregnancy outcome. This work shows the introduction of NIPD for confirmation of PGD results in eight pregnancies. In those pregnancies referred to PGD for an X-linked disorder (six out of eight), fetal sex determination in maternal blood was performed to confirm fetal sex. One pregnancy referred to PGD for Marfan syndrome and one referred for Huntington disease (HD) were also analyzed. In seven out of eight cases, PGD results were confirmed by NIPD in maternal blood. No results were obtained in the HD pregnancy. NIPD in PGD pregnancies can be a reliable alternative for couples that after a long process feel reluctant to undergo PD due to the risk of pregnancy loss.

## 1. Introduction

Preimplantation Genetic Diagnosis (PGD) allows performing a genetic diagnosis of the embryo to select those healthy embryos for uterine transfer. As an alternative to Prenatal Diagnosis (PD), PGD increases the chance of having a healthy child in those couples at high risk of transmitting a genetic disease, including monogenic disorders and imbalances due to chromosomal rearrangements [1]. A PGD cycle entails an ovaric stimulation, oocyte fertilization by IVF, embryo culture, embryo biopsy, genetic analysis and embryo transfer to the utero. Newborn birth success rate following PGD for monogenic disorders is around 20% [1] therefore, in a great percentage of cases, pregnancy has been achieved after a variable number of IVF cycles. Hence, the PGD process is usually long and very stressful for these couples.

Prenatal diagnosis is recommended in pregnancies resulting from PGD. However, PGD couples are unwilling to undergo PD due to their difficulties in conceiving and the risk of fetal loss associated with invasive prenatal testing. Circulating cell-free fetal fetal DNA (ccffDNA) in maternal blood allows non-invasive prenatal diagnosis (NIPD) without risk to the fetus and the mother. Only 10% of DNA present in maternal plasma is fetally derived; the remaining 90% is maternal DNA [2]. Thus far, NIPD has initially been focused on the analysis of *de novo* or paternally inherited traits to ensure their fetal origin, but application of new technologies are opening this field to maternally inherited alleles [3,4]. Sex and RhD determination were recently introduced into clinical practice [5,6,7,8].

Introduction of NIPD in the reproductive field is an interesting option to be offered not only to PD patients but also to PGD patients. In 2010, NIPD was considered as a confirmatory tool for PGD pregnancies in the ESHRE Best Practice Guidelines (European Society of Human Reproduction and Embryology) [9].

In this work, application of NIPD for confirmation of PGD results is described in eight pregnancies: six at-risk of an X-linked disorder, one at-risk of Marfan syndrome and one of Huntington disease.

## 2. Experimental Section

Twenty milliliters of maternal blood were collected and processed as previously described [8]. DNA was extracted from 1000 μL of plasma by using MagNA Pure Compact (Roche Diagnostics, Mannheim, Germany) and the MagNA Pure Compact Nucleic Acid Isolation Kit I Large Volumen (Roche Diagnostics, Mannheim, Germany).

### 2.1. Fetal Sex Determination in Maternal Blood

A total of 6 couples at risk of an X-linked disorder, in which the female was the carrier, had a pregnancy after IVF-PGD. The reasons for referral were: Choroideremia [2], Duchenne Muscular Dystrophy [2], Hemophilia [1] and X-linked Inmunodeficiency [1]. In these 7 pregnancies, fetal sex determination in maternal blood was performed in the first trimester of gestation in order to confirm fetal sex. Fetal sex determination was carried out by Real-Time PCR following the previously reported protocol [8].

### 2.2. Study of the Paternal Alleles in Maternal Plasma

A total of 2 couples at risk of a monogenic disorder with a singleton pregnancy after IVF-PGD were studied. The referral reasons were: Marfan syndrome and Huntington Disease. In both cases, the father of the fetus was carrier of the mutation.

Additionally to the maternal blood collection, ten milliliters of peripheral blood from the father were collected in these two couples. Paternal genomic DNA (gDNA) was extracted from 350 μL of peripheral blood by the use of the BioRobot EZ1 using the DNA Blood protocol (QIAGEN, Hilden, Germany).

#### 2.2.1. Marfan Syndrome

Two maternal blood samples were collected: at 11 and 13 weeks of gestation. For NIPD, both a direct analysis of the mutation and a haplotype analysis were performed. To establish the limit of detection of the protocol for the paternal change, a batch of artificial mixtures with decreasing amounts of paternal DNA in maternal DNA were created (10%, 5%, 2%).

##### 2.2.1.1. Direct Analysis of the Paternal Mutation

The paternal mutation was c.7636G>A; p.Gly2546Arg in the exon 62 of the *FBN1* gene. Analysis of the paternal change was carried out in 8 μL of plasma DNA and 1 μL of DNA from control genomic DNAs (the artificial mixtures, positive (paternal) and negative (maternal) DNAs), 10 pmol of each oligo (FBN1-F/FBN1-R) (Table 1), 2X Qiagen Multiplex Master Mix (QIAGEN, Hilden, Germany) and 10 × Buffer Q. PCR conditions were: an initial denaturation of 15′ followed by 96°15′′, 58°1′30′′, 72°1′ for 10 cycles and 94°15′′, 58°1′30′′, 72°1′ for 30 cycles and a final extension of 72°15′. PCR products were purified by using the columns (EZNA Cycle Pure Kit, OMEGA, Bio-tek, Norcross, GA, USA). Six microliters of purified PCR product from the plasma sample and 3 μL from the control DNAs were used as template for the second round of PCR (minisequencing). The reaction was carried out with 5 pmol of FBN1-S oligo following the manufacturer’s recommendations for 35 cycles (Table 1). Excess ddNTPs were removed by SAP enzyme (USB) and analyzed in an ABI3130xl Genetic Analyzer (Life Technologies, Foster City, CA, USA).

##### 2.2.1.2. Haplotype Analysis

As in the PGD cycle, MTS2 and MTS4 intragenic markers were studied. PCR conditions were similar as those used in the first round of PCR for the analysis of the paternal mutation. PCR products were analyzed in an ABI3130xl Genetic Analyzer (Life Technologies, Foster City, CA, USA).

#### 2.2.2. Huntington Disease (HD)

Four maternal blood samples were collected: at 8, 10, 12 and 24 weeks of gestation. CAG repeats and three STRs flanking *HTT* gene were analyzed (D4S126, D4S127 and I1CAHD) (Table 1). Because of previous personal experience in NIPD of HD [10,11,12,13], no artificial mixtures were studied. PCR were carried out as previously reported [13].

**Table 1:** Table with oligos sequences used in the study.

Disease/Case	Paternal Mutation/STR	Oligos Name	Oligos Sequence (5′ → 3′)
Marfan Syndrome	c.7636G>A (*FBN1* gene)	FBN1-F	5′-GCCCCCACTGCTTCTCA-3′
		FBN1-R	5′-CCTCCACAAGGATTCACCAG-3′
		FBN1-S	5′-CATTTGCCAGAACACTCCT-3′
	**MTS2** [14]	MTS2-F	5′-GTAGTTGTTATCTTGCAGA-3′
		MTS2-R	5′-CTGCCCTCTAGGACTCTAAG-3′
	**MTS4** [14]	MTS4-F	5′-GATGTCCCTATTGCCATCACCAC-3′
		MTS4-R	5′-CCTGTGCAGGGTAAGACAAG-3
Huntington disease	CAG repeats (*HTT* gene)	HTT-F	5′-ATGGCGACCCTGGAAAAGCTGATGAA-3′
		HTT-R	5′-GGCGGTGGCGGCTGTTGCTGC-3′
	**I1CAHD**	I1CAHD-F	5′-TATGCCACTACACTACAACCTGGGC-3′
		I1CAHD-F	5′-ACCAGCATGTGGTATTGTCAAAGTG-3′
	**D4S126**	D4S126-F	5′-GGATCCTGTCACTGTACT-3′
		D4S126-R	5′-GTTTCTTTGCTTAACCAGTTTGACCATGAGG-3′
	**D4S127**	D4S127-F	5′-CCTCTGTTTGCAATCCATTT-3′
		D4S127-R	5′-GTCCCTTGCATGCCCTGGCT-3′

## 3. Results and Discussion

When a pregnancy is confirmed after a Preimplantation Genetic Diagnosis (PGD) cycle, Prenatal Diagnosis (PD) is always recommended due to the associated diagnostic failure rate. In a great percentage of cases, pregnancy has been achieved after a variable number of IVF cycles and pregnant women usually reject invasive procedures because of the risk of pregnancy loss.

In the past, the confirmation of the diagnosis could only be performed by conventional prenatal diagnosis. However, the presence of ccffDNA in maternal blood opens a new alternative to these patients. The introduction of NIPD in the reproductive field is an interesting option to be offered not only to PD patients but also to PGD patients. In 2010, the European Society of Human Reproduction and Embryology (ESHRE) already considered NIPD as a confirmation tool in PGD pregnancies in their Best Practice Guidelines [9].

### 3.1. Fetal Sex Determination in Maternal Blood

The first application of NIPD introduced in the clinical practice was fetal sex determination. This non-invasive approach to the fetus is currently used in the management of pregnancies at-risk of sex-linked disorders. The incorporation of the fetal sex determination in maternal blood in the clinical practice in 2008 in our centre has caused a reduction of invasive obstetric procedures in around 50% of pregnancies at risk of a sex-linked disorder [8]. In the present work, confirmation of fetal sex in six PGD pregnancies with different referral reasons: Choroideremia, Duchenne Muscular Dystrophy, Hemophilia and X-linked Immunodeficiency is shown. NIPD of fetal sex revealed five female, and one male, fetuses. Fetal sex was concordant with transferred embryo sex in all six cases. In all those pregnancies in which a female fetus was confirmed, no further conventional PD was required.

**Figure 1:**
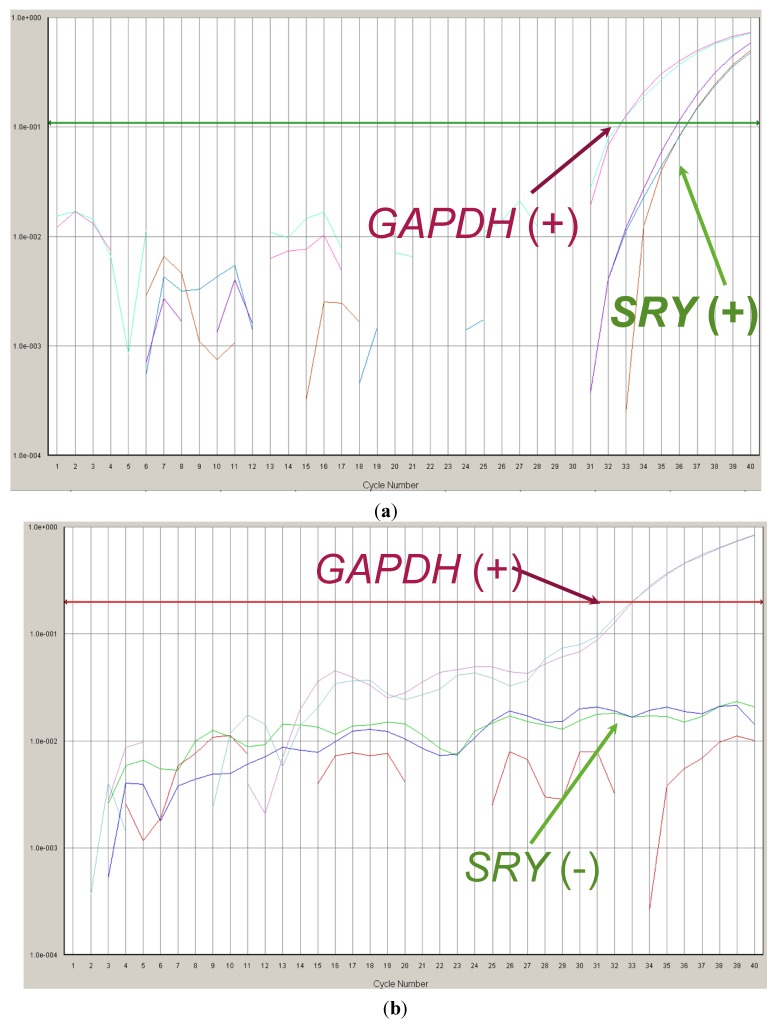
Analysis of *SRY* and *GAPDH* genes by Real-Time PCR in ccfDNA collected from maternal blood. Fetal sex determination is based on the presence/absence of the *SRY* gene. (**a**) Positive amplification for *SRY* and *GAPDH*. These results are associated to a male fetus; (**b**) Positive amplification for *GAPDH* and no amplification of *SRY* gene are associated to a female fetus.

The only male fetus determined corresponded to the X-linked immunodeficiency case (Figure 1a). In this case, two different embryos were transferred (female and healthy male) but only one (the male embryo) progressed. Since we were not able to offer NIPD for diagnosis of maternally inherited defects, the couple underwent conventional PD in order to confirm the healthy status of the male fetus. Finally, conventional PD confirmed PGD and NIPD results.

In case of PGD pregnancies at risk of a sex-linked disorder, fetal sex determination in maternal blood can be considered as a first step to validate the PGD results. It can reduce the need for further invasive prenatal testing. In case of a fetus with the at-risk gender (normally males), NIPD of the maternal defect is more challenging than the study of paternal alleles and it is still very limited within the clinical practice. We are currently working on the validation of new technologies (Digital PCR) that allow us to widen NIPD to the analysis of both paternal and maternal alleles [15].

However, NIPD of paternally inherited defects has been widely explored and there are many publications reporting the prenatal diagnosis of different diseases with a paternal origin [13,16,17,18,19,20,21,22].

### 3.2. Study of Paternal Alleles/Mutations in Maternal Plasma

In the present work, NIPD of two PGD pregnancies referred for Marfan syndrome and Huntington disease are described. In both cases, the pathologies have an autosomal dominant inheritance pattern and a paternal origin. NIPD of the paternal defect was performed in DNA from maternal plasma in order to confirm PGD results.

#### 3.2.1. Marfan Syndrome

In this case, the pregnancy came from a vitrified embryo. Two maternal plasma samples were analyzed: at 11 and 13 weeks of gestation. In order to establish the detection limit of the protocol designed, a panel of artificial mixtures of paternal DNA in maternal DNA was analyzed. Since the percentage of ccffDNA in maternal blood is around 9%–20%, a protocol with a detection limit comprised within this range is needed. Analysis of the artificial mixtures showed that with the designed protocol we were able to detect the paternal defect in the 10%, 5% and 2% artificial mixtures. Therefore it was established that the detection limit was 2% and the robustness of the test was confirmed. When DNA from the two plasma samples collected was studied, the paternal defect was not observed in any of them, confirming the healthy status of the fetus. These results were supported by the MTS2 analysis which showed the presence of the paternal allele without the mutation in the maternal plasma samples. However, no paternal/fetal alleles were observed in the MTS4 results.

#### 3.2.2. Huntington Disease

HD is one of the diseases in which our group has more experience in NIPD [10,11,12,13]. The experience in our lab with NIPD of HD comprises 25 cases with a diagnostic accuracy of 68% and a non-detection rate of 28% [23]. Herein, NIPD of a PGD gestation referred for HD diagnosis is shown. Four maternal plasma samples were collected but none paternally inherited fetal allele was detected with any of the three markers studied. Previous experience in NIPD of HD shows a non detection rate around 28% [23]. NIPD of HD is basically based on analysis of polymorphic regions (STRs) by QF-PCR. In our experience, NIPD by analysis of STRs is not as successful as other strategies like minisequencing or Real-Time PCR. In case of HD, it is also important to consider the number of the CAG repeats of the mutated allele. Since cfDNA fragments have been reported to be around 313 bp [24], amplification of larger fragments can increase the non-detection rate. In this particular case, non-detection of the paternal/fetal alleles was also observed in NIPD of a previous pregnancy of this couple. This phenomenon could also be related to lower amounts of cfDNA in maternal blood because some unknown physiological conditions of this particular pregnant woman.

Even though no results were obtained from the NIPD study, patients did not undergo conventional PD to confirm PGD results.

As shown in this work, analysis of paternally inherited fetal alleles in maternal blood can be useful for confirmation of PGD results. However, this approach shows two main limitations: (1) detection of maternally inherited disorders; and (2) non-detection of fetal alleles. We have just incorporated the digital PCR technology, a more sensitive technology that will help to improve our diagnosis either by: (1) helping in the detection of maternal alleles, thus being able to diagnose maternally inherited diseases; and (2) increasing the sensitivity rate and having less “non-detections”.

## 4. Conclusions

NIPD in PGD pregnancies represents an alternative for confirmation of PGD results for couples that, after a long IVF process, feel reluctant to undergo conventional invasive PD due to the risk of pregnancy loss. The incorporation of more sensitive techniques (Next Generation Sequencing and Digital PCR) is expanding the scope of non-invasive diagnosis [3,4].
